# The SP1-12LOX axis promotes chemoresistance and metastasis of ovarian cancer

**DOI:** 10.1186/s10020-020-00174-2

**Published:** 2020-05-06

**Authors:** Qi Zhang, Guifang Yan, Juan Lei, Yu Chen, Ting Wang, Juan Gong, Yong Zhou, Huakan Zhao, Hao Chen, Yu Zhou, Lei Wu, Jiangang Zhang, Xiao Zhang, Jingchun Wang, Yongsheng Li

**Affiliations:** 1 Clinical Medicine Research Center, Xinqiao Hospital, Army Medical University, Chongqing, 400037 China; 2 Institute of Cancer, Xinqiao Hospital, Army Medical University, Chongqing, China; 3 Chongqing Weisiteng Biotech Translational Research Institute, Chongqing, China; 4 Department of Medical Administration, Xinqiao Hospital, Army Medical University, Chongqing, China

**Keywords:** SP1, Lipoxygenase, Chemoresistance, Metastasis, Ovarian cancer

## Abstract

**Background:**

Ovarian cancer is the most lethal gynecologic cancer. Chemoresistance, especially platinum-resistance, is closely related to metastasis of ovarian cancer, however, the molecular basis by which links chemoresistance and metastasis remains vague. Disordered arachidonic acid (AA) metabolism has been shown to play an important role in the advanced ovarian cancer. This study aimed to explore the underlying mechanism involving eicosanoid metabolism that controlling chemoresistance and metastasis of ovarian cancer.

**Methods:**

Cisplatin (DDP)-resistant SKOV3 (SKOV3-R) cells were constantly induced. Ultra-high-performance liquid chromatography tandem mass spectrometry (UPLC-MS/MS) was performed to determine the AA metabolism in SKOV3 and SKOV3-R cells. Half maximal inhibitory concentration (IC50) and percentage of cell viability were tested using cell counting kit 8 (CCK-8). Realtime quantitative PCR (qPCR) and immunohistochemistry (IHC) were used to evaluate indicated genes and proteins respectively. Bioinformatic analysis and chromatin immunoprecipitation (ChIP) were performed to predict and identify the co-transcription factor of interest genes. Tumor growth and metastasis in the liver were assessed with nude mice by subcutaneously injection of SKOV3-R cells.

**Results:**

SKOV3-R cells expressed higher multidrug resistance-associated proteins (MRPs) MRP1 and MRP4. They showed enhanced metastatic ability and produced increased AA-derived eicosanoids. Mechanistically, MRPs, epithelial mesenchymal transition (EMT) markers Snail and Slug, as well as key enzymes involved in AA-metabolism including 12-lipoxygenase (12LOX) were transcribed by the mutual transcription factor SP1 which was consistently upregulated in SKOV3-R cells. Inhibition of SP1 or 12LOX sensitized SKOV3-R cells to DDP and impaired metastasis in vitro and in vivo.

**Conclusion:**

Our results reveal that SP1-12LOX axis signaling plays a key role in DDP-resistance and metastasis, which provide a new therapeutic target for ovarian cancer.

## Introduction

The lethality of ovarian cancer ranks the first in gynecologic cancers, and the majority of patients with ovarian cancer are diagnosed at the advanced stage at their first clinical evaluation (Torre et al. 2018). The regular therapy for ovarian cancer is cytoreductive surgery *plus* platinum-based chemotherapy (Ledermann et al. 2018). However, the platinum-resistance and high metastatic activity limited the efficacy of platinum-based chemotherapy (Oza et al. 2019). Thus, uncovering the mechanisms of platinum-resistance and metastasis is crucial for developing effective treatments to improve the prognosis of patients with ovarian cancer.

The multidrug resistance-related proteins (MRPs) are well-known associated with chemoresistance of ovarian cancer (Surowiak et al. 2006). In addition to pumping chemotherapy drugs out, MRPs efflux various eicosanoids such as leukotriene B_4_ (LTB_4_), LTD_4_, and prostaglandin E_2_ (van de Ven et al. 2008), which are derived from arachidonic acid (AA). Recently, disordered AA metabolism was confirmed to play an important role in the advanced ovarian cancer (Freedman et al. 2007). Furthermore, chemoresistance in cancer is often accompanied by enhanced metastasis (Turley et al. 2015). However, the underlying mechanisms linking chemoresistance to metastasis and whether AA metabolites contribute to this linkage are not yet clear.

In our study, we established cisplatin (DDP)-resistant SKOV3 (SKOV3-R) ovarian cancer cells and aimed to explore the mechanism driving chemoresistance and metastatic activity of SKOV3-R cells. Our study suggests a potential therapeutic target for patients with chemoresistant and metastatic ovarian cancer.

## Materials and methods

### Clinical database

The expression of SP1 in platinum-sensitive and -resistant in ovarian cancer patient was analyzed using the Gene Expression Omnibus (GEO) database [GSE114206; National Center for Biotechnology Information (NCBI)/NIH, Bethesda, MD, USA]. The Cancer Genome Atlas (TCGA) database was used for survival comparisons between patients with low and high levels of SP1. For gene correlation analysis, the database was downloaded from the NCBI GEO databases GSE13876 containing 415 patients with ovarian cancer. The Pearson correlation coefficient was used to analyze the correlation between indicated genes.

### Animal experiments

Nude mice (female, 8 weeks) from the Chinese Academy of Medical Sciences (Beijing, China) were injected with SKOV3 or SKOV3-R (1 × 10^6^ cells each) cells subcutaneously. For tumor volume assessment, mice were treated with PBS or DDP (1 mg/kg) *plus* baicalein (Bai; 30 mg/kg) or mithramycin (MA) (0.5 mg/kg) intraperitoneally every 2 days from Day 30 and were sacrificed at Day 36. For metastatic liver observation, mice were treated with PBS or DDP (1 mg/kg) *plus* Bai (30 mg/kg) or MA (0.5 mg/kg) intraperitoneally every 2 days in the third week after subcutaneous injection with SKOV3 or SKOV3-R cells and mice livers were collected at Day 21. This study was carried out in accordance with the relevant guidelines approved by the Institutional Animal Care and Use Committee of Army Medical University.

### The induction of DDP-resistant SKOV3

**Fig. 1:**
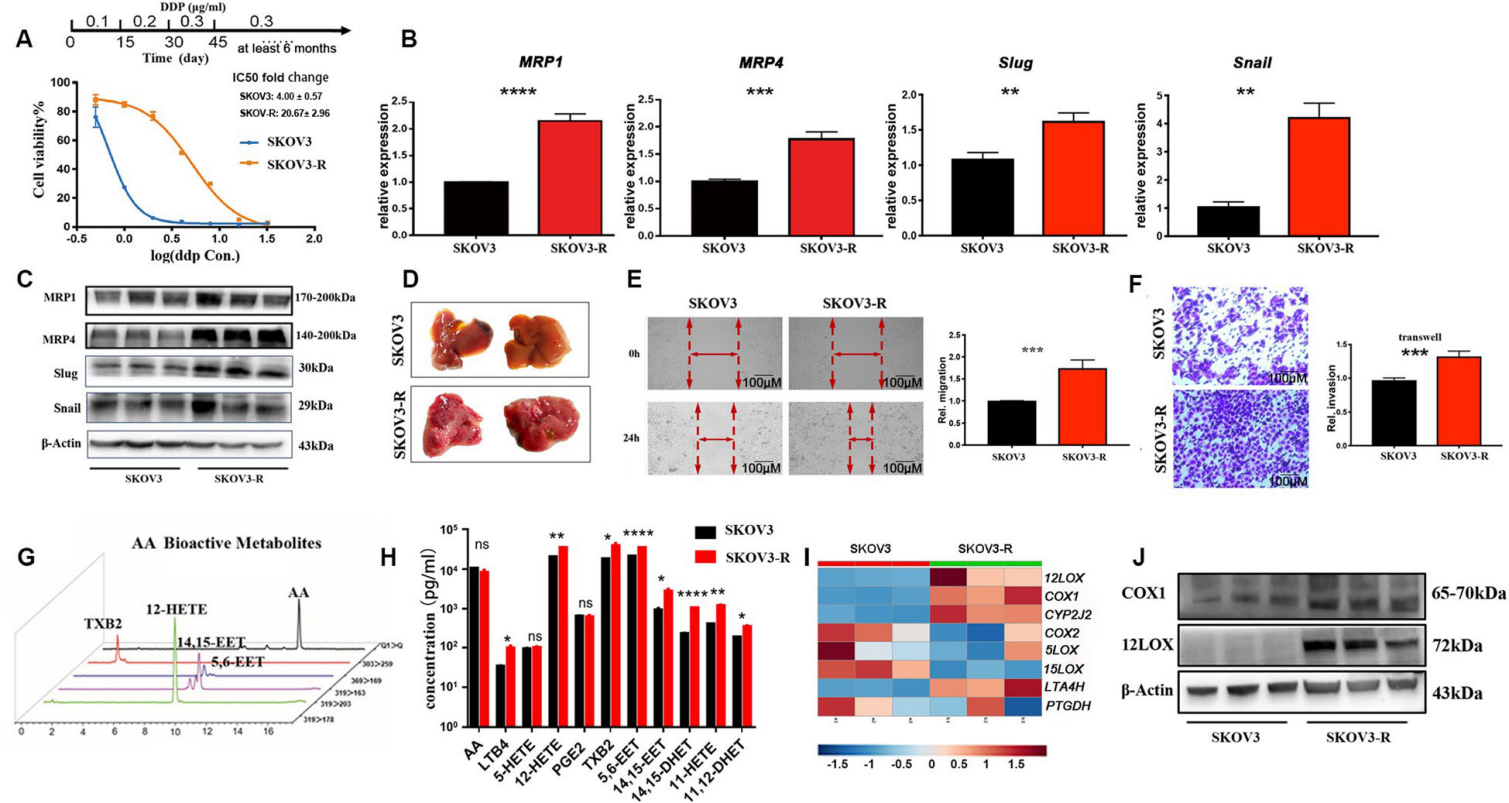
DDP-resistant ovarian cancer cells possess enhanced metastatic activity and arachidonic acid metabolism. **a** The procedure for the induction of DDP-resistant SKOV3 cells (upper panel); IC50 and the cell viabilities of SKOV3 and SKOV3-R with different concentrations of DDP treatment (lower panel). **b, c** The mRNA (**b**) and protein (**c**) levels of MRP1, MRP4, Slug and Snail in SKOV3 and SKOV3-R. **d** Liver metastasis in nude mice after SKOV3 or SKOV3-R subcutaneous transplantation. **e, f**. Migration and invasion of SKOV3 and SKOV3-R were tested by wound-healing (**e**) and transwell assays (**f**). **g** MRM chromatograms of AA, TXB_2_, 12-HETE, 5,6-EET, and 14,15-EET. **h** The supernatant levels of LTB_4_, 5-HETE, 12-HETE, PGE_2_, TXB_2_, 5,6-EET, 14,15-EET, 14,15-DHET, 11-HETE, and 11,12-DHET in SKOV3 and SKOV3-R. **i** The heatmap for gene expression of *12LOX*, *COX1*, *CYP2J2*, *COX2*, *5LOX*, *15LOX*, *LTA4H* and *PGDH*. **j** the protein levels of 12LOX and COX1 were assessed by western blotting. Data are expressed as mean ± SEM of 4 independent experiments. ^*^P < 0.05, ^**^P < 0.01, ^***^P < 0.001, ^****^P < 0.0001

Mouse SKOV3 cells from ATCC (Manassas, VA, USA) were cultured in RPMI 1640 (Gibco™/Thermo Fisher Scientific, Waltham, MA, USA) with 10% FBS (Gibco) and 1% penicillin-streptomycin (Gibco). Cells were routinely verified Mycoplasma-free using MycAway™-Color One-Step Mycoplasma Detection Kit (Yeasen Bio-technol) and the most recent date of testing was April 12, 2019. SKOV3 cells were induced by gradually increasing the concentration of DDP ranging from 0.1 to 0.3 μg/mL for 45 days and then these cells were maintained in 0.3 μg/ml of DDP constantly for at least 6 months as described in Fig. 1a (upper panel).

### Cell proliferation and cytotoxicity

A Cell Counting Kit-8 (CCK-8) (Dojindo, Kumamoto, Japan) was used to determine the cell viability and the half maximal inhibitory concentration (IC50) value of SKOV3 and SKOV3-R exposed to DDP. The cell suspension (4000 cells/100 μL per well) was inoculated into a 96-well plate in a 5% CO_2_ incubator at 37 °C for 12 h. Each group was incubated with 200 μL of 1640 medium containing different doses of DPP for 48 h, and then was incubated with CCK-8 reagent for 2 h according to the manufacturer’s instructions. The OD values were determined at 450 nm using the microplate reader Varioskan FLASH (Thermo Fisher Scientific).

### Wound healing and transwell invasion assays

For the wound-healing migration assay, cells (1 × 10^6^/well) were seeded in 6-well plates containing 1640 medium with 10% FBS in a 5% CO_2_ incubator at 37 °C overnight. A continuous scratch wound was created by scratching a 10 μL pipette tip across the surface of the plate wells then cells were washed with PBS for three times. Cells were subsequently treated with DDP (0.3 μg/mL), MA (30 nM), and Bai (20 μM) for 24 h in 1640 medium. The area of a defined region within the scratch was measured using ImageJ software (NIH).

For invasion assays, transwell inserts (BD Biosciences, San Jose, CA, USA) with 8-μm pore size filters covered with Matrigel® were inserted into 24-well plates. The cells were serum-starved overnight and incubated in the upper chamber with 1640 medium [fetal bovine serum (FBS)-free] (2.5 × 10^5^ cells per insert) with different compounds at indicated concentrations and the 1640 medium supplemented with 20% FBS was added to the lower chamber. After 24 h of incubation at 37 °C, the non-invading cells that remained on the upper surface of the filter were removed gently by cotton swab and the cells that had traversed the filter and attached to the bottom membrane were fixed in methanol and stained with 0.2% crystal violet. The number of the invasive cells from triplicate chambers in randomly selected fields were counted in each experiment under Leica DMI3000B microscope (Leica Microsystems Inc., Buffalo Grove, IL).

### Quantitative reverse transcription PCR (RT-qPCR)

Total RNA was extracted with a kit purchased from Invitrogen/Thermo Fisher Scientific (Carlsbad, CA, USA) (no. 00490515) and transcribed into cDNA using Takara PrimeScript RT Reagent Kit with gDNA Eraser (Takara, Kusatsu, Japan). qPCR was performed using a CFX384 system (Bio-Rad, Hercules, CA, USA). The primer sequences were as follows:

β-actin-F GCGCGGCTACAGCTTCA; β-actin-R CTTAATGTCACGCACGATTTCC; MRP1-F TGCTCACTTTCTGGCTGGTA; MRP1-R ACAGGACAAGACGAGCTGAA; MRP4-F AAGTGAACAACCTCCAGTTCCAG; MRP4-R GGCTCTCCAGAGCACCATCT; 12 lipoxygenase (12LOX)-F CAACGTGATCCGAGGAGAGAA; 12LOX-R GTGTTCAGCAAGTGATACTGGA; cyclooxygenase 1 (COX1)-F CTCTGTGCCTAAAGATTGCCC; COX1-R GTCTCCATAAATGTGGCCGAG; cytochrome P450 2 J2 (CYP2J2)-F GAGCTTAGAGGAACGCATTCAG; CYP2J2-R GAAATGAGGGTCAAAAGGCTGT; COX2-F TGAGTGTGGGATTTGACCAG; COX2-R TGTGTTTGGAGTGGGTTTCA; 5LOX-F ACTGGCTGAATGACGACTGG; 5LOX-R CAGGGGAACTCGATGTAGTCC; PGDH-F TGCTTCAAAGCATGGCATAG; PGDH-R GGGTTTTTGCTTGAAATGGA; 15LOX-F TGTGAAAGACGACCCAGAGC; 15LOX-R GGTGACAAAGTGGCAAACCT; LTA4H-F CCACCATCCTTCCCTTAT; LTA4H-R AAACAATCGTCCGCAAAT; SP1-F TGGCAGCAGTACCAATGGC; SP1-R CCAGGTAGTCCTGTCAGAACTT; Snail-F ACTGCAACAAGGAATACCTCAG; Snail-R GCACTGGTACTTCTTGACATCTG; Slug-F CGAACTGGACACACATACAGTG; Slug-R CTGAGGATCTCTGGTTGTGGT.

### Western blotting

Samples were lysed using RIPA lysis buffer with phenylmethylsulphonyl fluoride and quantified using a BCA assay (catalog no. P0068; Beyotime, Jiangsu, China). The primary antibodies include MRP1 [catalog no. 14685S, Cell Signaling Technology, (CST) Danvers, MA, USA], MRP4 (catalog no. 12705, CST), SP1 (catalog no. 9389, CST), 12LOX (catalog no. ab167372, Abcam, Cambridge, UK), Snail (catalog no. 3879, CST), Slug (catalog no. 9585, CST) and β-Actin (catalog no. 3700, CST). After 5 washes with TBST, the membranes were incubated for 60 min with horse radish peroxidase-conjugated secondary antibodies (catalog no. A0562, Beyotime; 1:5000 dilution in TBST). The bands were visualized with an enhanced chemiluminescence (ECL) plus western blotting detection kit (catalog no. P0018–2, Beyotime).

### AA metabololipidomics

Lipids were extracted and isolated as described previously (Zhang et al. 2018a). Briefly, samples were purified through an SPE column (500 mg × 6 mL Sep-Pac C18; Waters, Milford, MA, USA). The targeting components were next dried under N_2_ and residues were reconstituted utilizing a methanol and water mixture (v/v, 1:1). A final 10 μL aliquot of each sample was introduced into an AQUITY UPLC (Waters) (BEH-C18 2.1 × 100 mm, 2.1 × 50 mm, 1.7 μm, Waters)-AB SCIEX QTRAP 6500 system (ESI mode). Moreover, the column, gradient program, and multiple reaction monitoring (MRM) were optimized during the monitoring processes (Zhang et al. 2018a). Analyst® 1.6.2 and MultiQuant™ software (Applied Biosystems/Thermo Fisher Scientific, Foster City, CA, USA) were used to acquire and quantify all lipids.

### Treatment with siRNAs

The siRNA of SP1 and control siRNA were purchased from RIBOBIO (no. SIGS0003589–1, Guangzhou, China). These siRNAs were combined with Lipofectamine™ 2000 Transfection Reagent (no. 11668019, Thermo Fisher Scientific Inc., USA) and diluted to a final concentration of 10 nM SP1-siRNA and negative control (siNC) in Opti-MEM™ I Reduced Serum Medium. After 72 h transfection, cells were used for functional studies.

### The bioinformatic prediction of transcription factor and ChIP

The bioinformatic prediction of the transcription factor was performed using JASPAR 2016, an open database of transcription factor binding profiles (Mathelier et al. 2016). ChIP assays were performed using a ChIP Kit (Abcam). Briefly, chromatin from cells was cross-linked with 1% formaldehyde for 10 min at 20 °C, sheared to an average size of 500 bp, and immunoprecipitated with an ATF4 antibody (Abcam). ChIP assay primers ABCC1 [catalog no. GPH004786(−)18A, Qiagen, Hilden, Germany], ABCC4 [catalog no. GPH017397(−)01A, Qiagen], ALOX12 [catalog no. GPH005277(−)01A, Qiagen] were used to amplify a proximal promoter region containing the SP1 putative binding element. Each immunoprecipitated DNA sample was quantified using qPCR and all ChIP-qPCR signals were normalized to the input.

### Histological analysis of liver metastasis and immunohistochemistry

The mice liver specimens were fixed in 10% neutral-buffered formaldehyde immediately for over 24 h and then were dehydrated in isopropyl alcohol, followed by clearing of alcohol by xylene. Subsequently, the dehydrated specimens were embedded in paraffin. Paraffin specimens were stained with hematoxylin and eosin (H&E)) or were performed immunohistochemistry (IHC) for SP1, MRP1, 12LOX, and Snail. The mean density (IOD/area) were detected in different positive areas of liver specimens using Image-pro Plus 6.0 software. Liver metastatic burden was calculated by the quotient of IOD values in the average area of tumor lesions divided by the values in the total area of liver section. The results were analyzed and normalized against expression in PBS group. Hepatic lesions were digitally marked and tumor number of per section was assessed by the ZEN imaging software.

### Flow cytometry

The single-cell suspension was prepared, fixed, and penetrated with a Fixation/Permeabilization Kit (BD Biosciences) according to the manufacturer’s instructions, and then was stained with Ki67 antibody (Invitrogen). The FACS Canto II system (BD Biosciences) and FlowJo software (Tree Star, Ashland, OR, USA) were used to determine the fluorescence.

### Statistical analysis

Statistical analyses were performed using GraphPad Prism 8.0 (GraphPad Software, Inc., La Jolla, CA, USA). All data were analyzed using either one-way ANOVA or two-tailed unpaired Student’s t-test and represented as the mean ± SEM. *P* ≤ 0.05 was considered statistically significant. Gene data of the key enzymes in AA metabolism pathway were subjected to Heatmaps using MetaboAnalyst 4.0 (http://www.metaboanalyst.ca/).

## Results and discussion

### SKOV3-R cells possessed enhanced metastatic activity and produced higher AA-derived eicosanoids

SKOV3 cells were treated with gradually increasing concentrations of DDP for 45 days to establish a DDP-resistant SKOV3-R cell line (Fig. 1a, upper panel). The CCK-8 assay and increased expression levels of MRP1 and MRP4 confirmed the DDP-resistance capacity of the SKOV3-R cells (Fig. 1a and b). Moreover, the epithelial mesenchymal transition (EMT) markers including Snail and Slug were significantly boosted in SKOV3-R cells, compared with that in SKOV3 cells (Fig. 1b and c).

To validate the enhanced metastatic activity of DDP-resistant ovarian cancer cells in vivo, we employed a nude mouse model. The mice injected with SKOV3-R cells showed macroscopic and widespread metastasis in the liver, while the liver of SKOV3 group didn’t show obvious metastases (Fig. 1d). We also used wound-healing and transwell assays to assess migration and invasion in vitro. SKOV3-R showed strikingly enhanced migration and invasion activities (Fig. 1e and f). These results demonstrate that SKOV3-R cells exhibit higher DDP-resistance and metastatic activity than SKOV3 cells.

Given that MRPs also pump AA-derived eicosanoids including LTB_4_ and PGE_2_ out to the extracellular matrix (Capannolo et al. 2015; Chen et al. 2017; Rius et al. 2008), the levels of eicosanoids could be considered activity indicators of MRPs. We used UPLC-MS/MS to analyze the AA metabolome in the supernatants of cells. The results showed that various metabolites including LTB_4_, 12-HETE, TXB_2_, 5,6-EET, 14,15-EET, 14,15-DHET, 11-HETE, and 11,12-DHET were much higher in SKOV3-R cell supernatants than in the SKOV3 group (Fig. 1g-h).

We next assessed key enzymes involved in the biosynthesis of the above altered lipids. We found that 12LOX, COX1, and CYP2J2 were markedly upregulated in the SKOV3-R cells (Fig. 1i and j). Together, these findings demonstrate that in addition to expressing higher MRPs, SKOV3-R cells produce higher AA-derived eicosanoids.

### SP1 is the mutual transcription factor of MRP1, MRP4, snail, slug, 12LOX, COX1, and CYP2J2

**Fig. 2:**
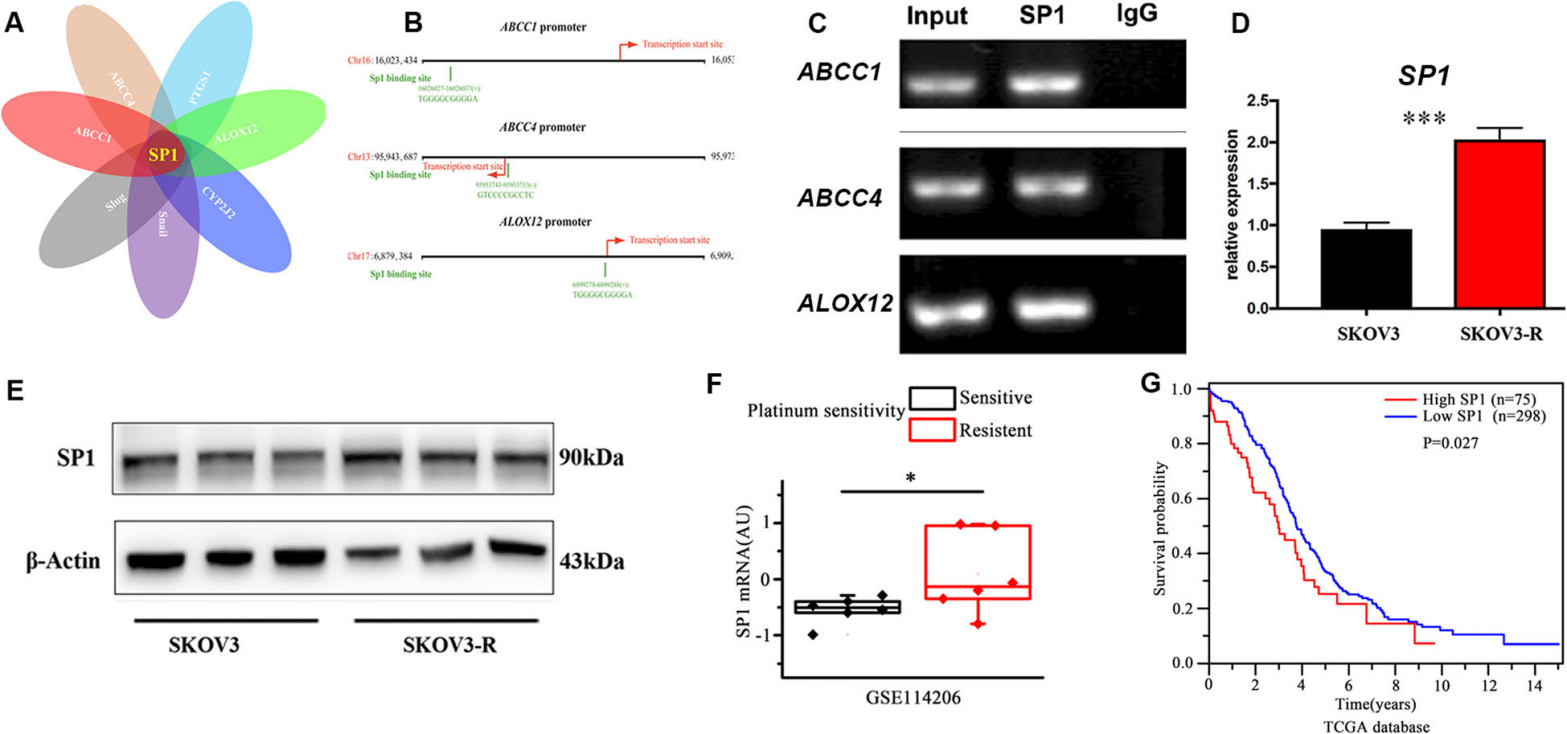
Identification of SP1 as the mutual transcription factor of MRPs and 12LOX. **a** Bioinformatic analysis of transcription factors of *MRP1*, *MRP4*, *Snail*, *Slug*, *12LOX*, *COX1*, and *CYP2J2*. **b** SP1 binding sequences in the promoters of *MRP1* (*ABCC1*), *MRP4* (*ABCC4*), and *12LOX* (*ALOX12*). **c** ChIP assay of SP1 binding on the promoters of *MRP1* (*ABCC1*), *MRP4* (*ABCC4*), and *12LOX* (*ALOX12*). **d, e** The mRNA (**d**) and protein (**e**) expression levels of SP1 in SKOV3 and SKOV3-R cells. Data in (**d**) are expressed as mean ± SEM of 4 independent experiments. ^***^P < 0.001. **f** The unpaired Student’s *t* test was used to analyze the expression of SP1 in the GEO database (GSE114206). Data are expressed as mean ± SEM and ^*^P < 0.05. **g** Patient survival data were obtained from TCGA database and overall survival probability was then calculated using the Kaplan-Meier method and the differences in survival curves were analyzed using the log-rank test. *P*-values of 0.05 or less were considered significant

To explore the underlying mechanisms that link chemoresistance, metastasis, and AA metabolism in ovarian cancer, we analyzed the potential co-transcription factors of MRPs, EMT markers (Snail and Slug), and the enzymes identified above via bioinformatics. We found that the promoters of *MRP1* (*ABCC1*), *MRP4* (*ABCC4*), *Slug*, *Snail*, *CYP2J2*, *12LOX* (*ALOX12*), and *COX1* (*PTGS1*) shared a mutual transcription factor SP1 (Fig. 2a and b).

SP1 is highly expressed in various cancer including gastric, breast, lung and glioma, which is associated with poor prognosis (Beishline and Azizkhan-Clifford 2015). SP1 contributes to the proliferation and invasion in hepatocellular carcinoma and astrocytoma (Chen et al. 2019; Zhang et al. 2018b). Previous evidence also showed that SP1 was an important modulator in ovarian cancer and deleting SP1 reduced the risk of ovarian cancer (Knappskog et al. 2011). In addition, SP1 was reported to participate in the transcriptional regulation of COX1, CYP2J2, Slug, and Snail (Taniura et al. 2002; Spiecker et al. 2004; Choi et al. 2007; Lee et al. 2018). However, the chemoresistance and metastasis mechanisms regulated by SP1 in ovarian cancer remain unclarified. Our ChIP assay verified that SP1 was involved in the transcription of *MRP1*, *MRP4*, and *12LOX* (Fig. 2c). SP1 was significantly upregulated at both the gene and protein levels in SKOV3-R cells (Fig. 2d and e). We also analyzed the expression of SP1 in ovarian cancer patients using the human GEO database (GSE114206), and found that SP1 was highly expressed in platinum-resistant patients, compared with control subjects (Fig. 2f). In addition, the TCGA database analysis showed that SP1 expression negatively correlated with survival in ovarian cancer patients (Fig. 2g).

Taken together, these results indicate that SP1 is the mutual transcription factor of *MRP1*, *MRP4*, *Snail*, *Slug*, *COX1*, *12LOX*, and *CYP2J2*, which links platinum-resistance, metastasis, and AA metabolism in ovarian cancer.

### Targeting SP1 or 12LOX inhibited DDP-resistance and EMT of SKOV3-R in vitro

**Fig. 3:**
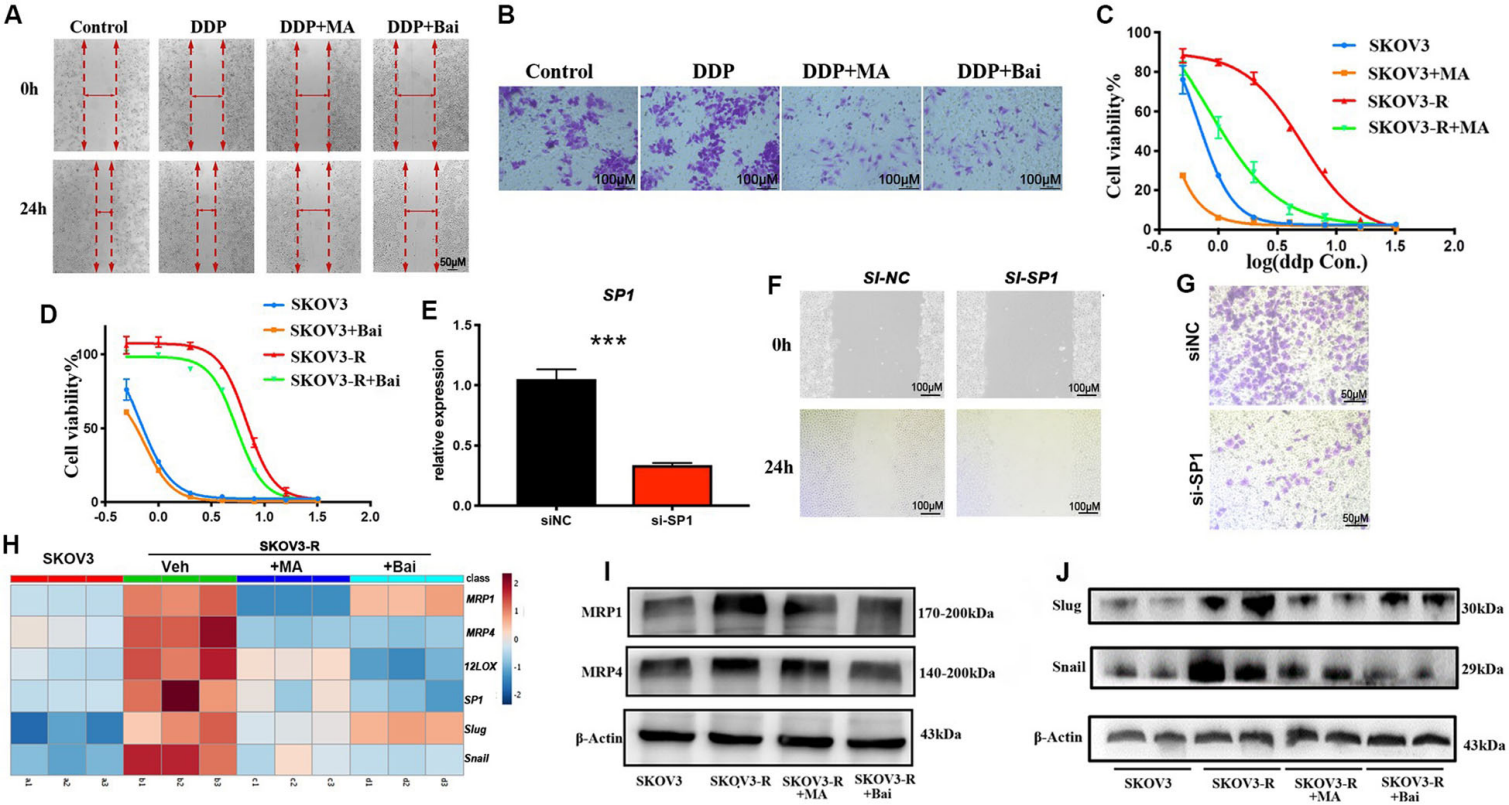
Inhibition of SP1 or 12LOX dampens metastasis and DDP-resistance of SKOV3-R cells in vitro. **a-d** After incubation with Bai (20 μM) or MA (30 nM) for 24 h, migration and invasion were tested by wound-healing assays (**a**) and transwell assays (**b**); the cell viability percentages were measured by CCK-8 assay (**c, d**). siNC denoted SP1 siRNA negative control, si-SP1 denoted SP1 siRNA. **e-g** After SKOV3-R cells were treated with these siRNAs, the gene expression levels of SP1 was evaluated by qPCR (**e**) and migration and invasion were tested by wound-healing assays (**f**) and transwell assays (**g**). **h-j** After administration with Bai (20 μM) or MA (30 nM) for 24 h, the heatmap for gene expression of *MRP1*, *MRP4*, *SP1*, *12LOX*, *Slug* and *Snail* (**h**) and the protein level of MRP1, MRP4, Slug and Snail were evaluated by western blotting (**i,j**). Data are expressed as mean ± SEM of 4 independent experiments. ^***^P < 0.001

Since the impacts of SP1 on the transcription of COX1 and CYP2J2 have been reported previously (Spiecker et al. 2004; Gibson et al. 2005), here we focused on the effect of SP1 and 12LOX on the DDP-resistance and metastasis of SKOV3-R cells. 12LOX plays an essential role in the cell survival and metastasis of ovarian cancer cells (Freedman et al. 2007; Seo et al. 2012). Intriguingly, 12LOX product 12HETE was also reported to induce Sp1 translocation to the nuclei (Garcia-Verdugo et al. 2012). We found that both mithramycin A (MA, the SP1 inhibitor) and baicalein (Bai, the inhibitor of 12LOX) significantly suppressed the migration and invasion of SKOV3-R cells (Fig. 3a and b). MA not only markedly enhanced the sensitivity of SKOV3-R cells to DDP, but also sensitized SKOV3 cells to DDP (Fig. 3c and d). Knockdown of SP1 significantly inhibited the migration and invasion of SKOV3-R (Fig. 3i and g), which was consistent with the results from MA treatment experiments. Moreover, the mRNA levels of *MRP1, MRP4,12LOX, Slug* and *Snail* were significantly downregulated after blocking SP1 or 12LOX (Fig. 3h-j). These results demonstrate that suppression of SP1 or 12LOX dampens EMT and DDP-resistance of SKOV3-R cells.

### Targeting SP1 or 12LOX attenuated metastasis and reversed DDP-resistance of SKOV3-R in vivo

**Fig. 4:**
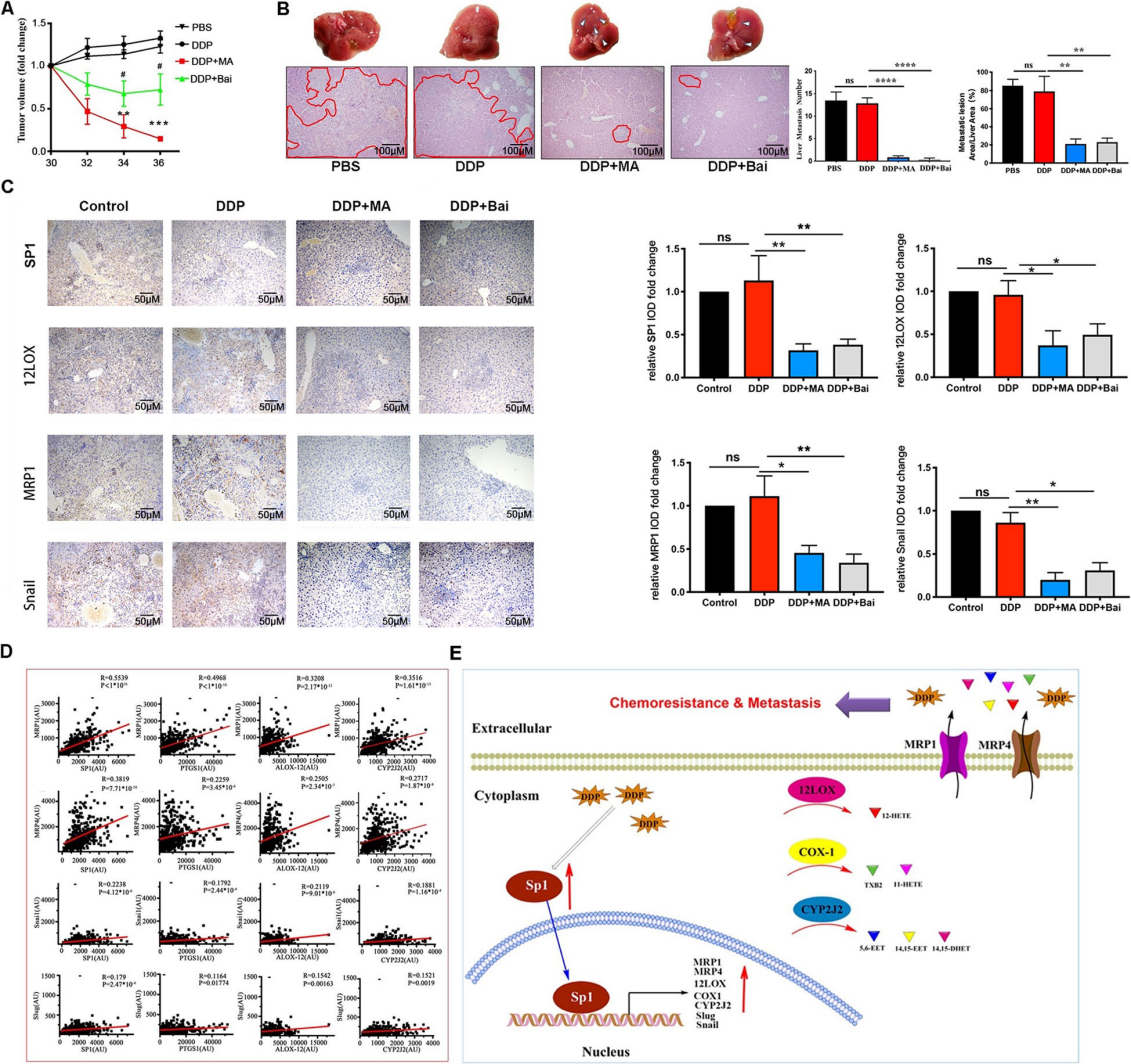
Blocking SP1 or 12LOX attenuates SKOV3-R tumor growth and reverses metastasis in vivo. **a** SKOV3-R cells (1 × 10^6^) were subcutaneously injected in nude mice, tumor reached ~ 300 mm^3^ at Day 30, then the mice were treated with PBS or DDP (1 mg/kg) or DDP (1 mg/kg) plus Bai (30 mg/kg) or MA (0.5 mg/kg) once every 2 days. The tumor volume was assessed until Day 36. **b** SKOV3-R cells (1 × 10^6^) were subcutaneously injected in nude mice. The mice were treated with PBS or DDP (1 mg/kg) plus Bai (30 mg/kg) or MA (0.5 mg/kg) every 2 days in the third week. Mice were sacrificed for assessment of liver metastasis at Day 21. The overall view of the livers, H&E staining and the metastasis numbers in live lesions (right panel) were determined. Data in (**b**) are expressed as mean ± SEM for 5 mice. **c** IHC of SP1, 12LOX, MRP1 and Snail in liver tissues in four groups including control (PBS), DDP, DDP *plus* MA, DDP *plus* Bai. IOD against control group in above groups were depicted in fold change. **d** The correlation between SP1, 12LOX, COX1, CYP2J2 with MRPs, Slug and Snail. Pearson correlation coefficient was used to analyze the correlation between *MRPs* and EMT markers with *SP1*, *12LOX*, *COX1*, and *CYP2J2* using the clinical database GSE13876. **e** Schematic diagram of SP1-driven chemoresistance and metastasis. Data are expressed as mean ± SEM for 5 mice. ^*^P < 0.5, ^**^P < 0.01, ^***^P < 0.001, ^****^P < 0.0001; DDP + MA vs. DDP; ^#^*P* < 0.05, DDP + Bai vs. DDP

To further explore the role of SP1 and 12LOX in regulating tumor growth and metastasis of DDP-resistant ovarian cancer in vivo, nude mice were subcutaneously injected with SKOV3-R cells and intraperitoneally injected with DDP (1 mg/kg), with/without MA (0.5 mg/kg) or Bai (30 mg/kg). Intriguingly, administration of MA and Bai both markedly inhibited the tumor volume compared to treatment with DDP alone (Fig. 4a). Both MA and Bai also significantly decreased the number of metastatic tumors and lesion in the liver compared with the PBS and DDP groups (Fig. 4b). We next used IHC to assess the expression of SP1, 12LOX, MRP1 and Snail in liver tissue, and found that MA and Bai inhibited these proteins (Fig. 4c).

In addition, the relationship between SP1-12LOX signaling with the indicated gene transcripts was assessed in the NCBI GEO clinical database (GSE13876) containing 435 ovarian cancer patients. In line with our above experiments, *SP1* and *12LOX* positively correlated with *MRP1*, *MRP4*, *Slug,* and *Snail*. We also examined the correlation of *COX1* and *CYP2J2* with *MRPs* and EMT-related genes in this database, our results suggesting that the expression of *COX1* and *CYP2J2* were also positively correlated with *MRP1*, *MRP4*, *Slug*, and *Snail*, respectively (Fig. 4d). Together, these results indicate that the SP1-12LOX axis contributes to DDP-resistance and metastasis of ovarian cancer (Fig. 4e).

## Conclusion

In summary, we identified a novel SP1-12LOX axis that linked DDP-resistance and metastasis in ovarian cancer cells. Our study provides the molecular basis for the inhibition of the SP1-12LOX axis as a potential therapeutic approach to improve the prognosis of patients with ovarian cancer.

## Data Availability

All data needed to evaluate the conclusions in the paper are present in the paper. Additional data related to this paper may be requested from the authors.

## Abbreviations

AA: Arachidonic acid
ABC: ATP-binding cassette
Bai: Baicalein
BSA: Bovine serum albumin
CCK-8: Cell counting kit 8
ChIP: Chromatin immunoprecipitation
COX: Cyclooxygenase
CYP2J2: Cytochrome P450 2 J2
DDP: Cisplatin
EMT: Epithelial mesenchymal transition
GST: Glutathione S-transferase family
HETE: Hydroxy eicosatetraenoic acid
IC50: Half maximal inhibitory concentration
LT: Leukotriene
LOX: Lipoxygenase
MA: Mithramycin A
MDR: Multidrug resistance
MRP: Multidrug resistance-associated protein
PBS: Phosphate buffered saline
PGE_2_: Prostaglandin E_2_
qPCR: Quantitative polymerase chain reaction
sES: Soluble epoxide hydrolase
SP1: Sp1 transcription factor
TXB2: Thromboxane B2
TBST: Tris-buffered saline containing 0.05% Tween 20
UPLC-MS/MS: Ultra-high performance liquid chromatography tandem mass spectrometry
